# A giant cell rich osteosarcoma of the proximal ulnar bone treated by elbow arthroplasty: A case report

**DOI:** 10.1016/j.ijscr.2019.04.017

**Published:** 2019-04-16

**Authors:** S.D. Cahayadi, A. Antoro, B. Swandika

**Affiliations:** Department of Orthopaedic and Traumatology, Persahabatan General Hospital, Jalan Persahabatan Raya No.1, 13230 East Jakarta, Indonesia

**Keywords:** Giant cell rich type, Osteosarcoma, Proximal ulnar bone, Elbow arthroplasty, Latissimus dorsi flap, Case report

## Abstract

**Introduction:**

Osteosarcoma is a malignant tumor of the bone. The treatment and prognosis have changed dramatically these days. Limb salvage surgery is a widely accepted alternative to amputation in patients with sarcoma. In most cases, the diagnosis of osteosarcoma is not difficult. We reported a rare location and a rare type of osteosarcoma case treated by elbow arthroplasty along with the challenging diagnostic and treatment.

**Case presentation:**

We reported 46 years old female complained of pain on her left elbow for 5 months. An x-ray showed lytic lesion on the medial side of the elbow while the MRI examination suggested a malignant bone tumor in the epi-meta-diaphyseal of left ulnar bone. Core biopsy showed a Giant Cell containing lesion. The histology of resected tumor and Ki67 staining confirmed the diagnosis of giant cell-rich type osteosarcoma.

**Discussion:**

Giant cell rich osteosarcoma is an uncommon variant of osteosarcoma which characterized by an abundance of osteoclastic giant cells and lack of tumor osteoid. Involvement of forearm in conventional osteosarcoma is extremely rare. This was the epidemiologic reason why the osteosarcoma was not the first diagnosis of our case. Being a very rare location of the osteosarcoma, the management poses a special challenge. They are the surgical technique, which is not well described, and the implant that need to be custom made.

**Conclusion:**

Giant cell rich osteosarcoma is difficult to diagnose because its histology and radiologic feature mimic a benign giant cell tumor.

## Highlights

• Osteosarcoma of the proximal ulnar bone is a malignant progressive bone tumors.
• Diagnosis of these cases needs a comprehensive history taking physical examination and additional diagnostic test.
• Limb salvage surgery using wide excision and reconstruction with elbow arthroplasty by our institution was a challenging procedure.
• Latissimus dorsi flap is important to close the defect left by wide excision.

## 1 Introduction

Osteosarcoma (OS) is a primary malignant bone tumor which affects around 3.4 per million people per year [1]. The tumor mostly occurs in children, adolescents, and young adults between the ages of 10–25 years; although, it can also affect people between 60–80 years of age and children younger than 5 years. [2]. Clinical presentation may vary from incidental finding to local pain, swelling or pathological fracture [3,4]. Plain radiographs show classic lytic or blastic lesions, with periosteal reaction, and destruction of the bone. The tumor might appear as hypo- or hyperintense lesion on MRI T1-weighted sequence, depending on the amount of adipose tissue; or hyperintense on T2-weighted sequence due to vascularity [5,6].

Histopathological examination serves as a golden diagnostic standard for osteosarcoma. In most cases, the diagnosis of osteosarcoma is not difficult. The presence of malignant osteoid matrix among malignant cells are pathognomonic [1]. Histologically, osteosarcoma can be classified as low-grade intramedullary osteosarcoma, high-grade surface osteosarcoma, telangiectatic osteosarcoma, conventional osteosarcoma, parosteal osteosarcoma, and periosteal osteosarcoma [1]. Conventional osteosarcoma is the most common type with an aggressive characteristic. They are predominant in the epimetadiaphysis of the long bone [5,7].

Traditionally, wide excisions and amputations are performed as a single treatment to treat osteosarcoma. Introduction of neoadjuvant chemotherapy in addition to conventional surgery effectively improve survival rate, especially if the response to the chemotherapy is adequate [8]. Following excision of local neoplasm, the focus of surgical treatment is in the restoration of the biomechanics of the adjacent joints [9,10]. In the osteosarcoma near the elbow joint, Van Isacker et al advocated elbow arthroplasty, because it allows for immediate restoration of the anatomy to preserve joint motion. So the patient still has a functional extremities and joint. We reported a rare location and a rare type of osteosarcoma case treated by elbow arthroplasty. The case has been reported in line with the SCARE criteria for better understanding [11].

## 2 Presentation of case

**Fig. 1:**
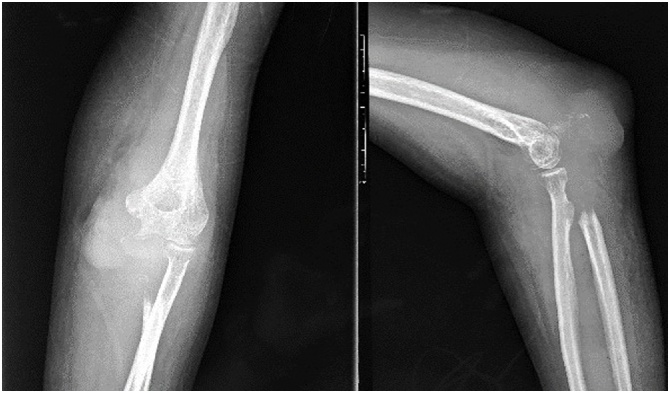
Pre operative x-ray of left elbow. There is lytic area in proximal of the ulnar bone.

**Fig. 2:**
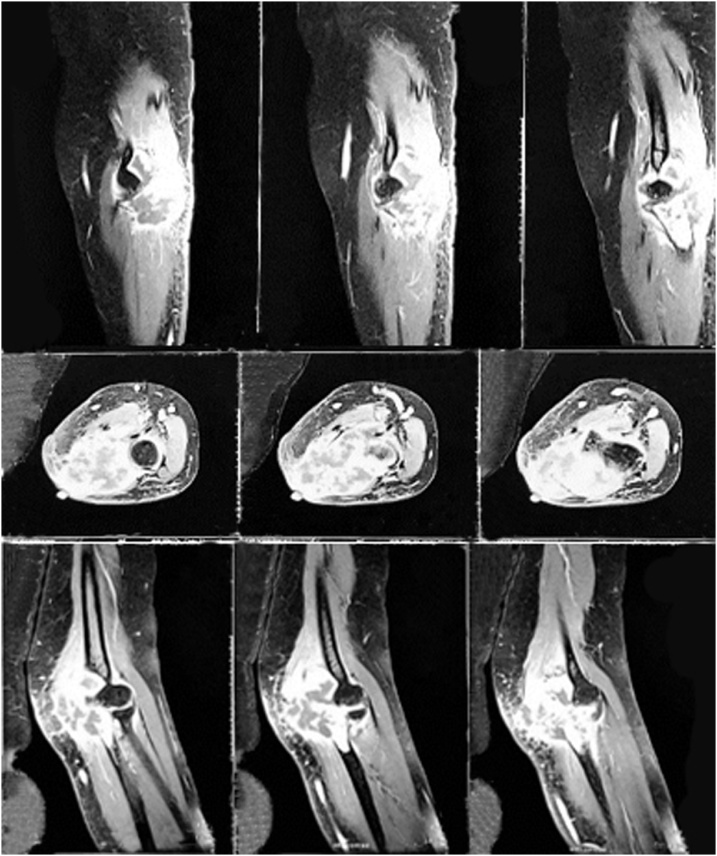
Pre operative MRI of right forearm. Hyperintense heterogen mass was seen at proximal ulnar bone that involves pronato teres, flexor digitorum superficialis, and fleksor carpi ulnaris muscles. The mass caused irregularity, necrotic area, and destruction of the proximal ulnar bone which suggested a malignant lesion.

Our patient was a 46 years old female complained of pain on her left elbow for 5 months. She went to a doctor and was given NSAID, but the pain was not relieved. A month later, she went to our hospital because of her elbow swelling and the pain which became severe. We did an elbow x-ray that shows a soft tissue mass on the medial side of the elbow, a radiolucent area within distal of the humeral and proximal of the ulnar bone (Fig. 1). We also found nodes at both lungs from thorax x-ray. MRI examination suggested a malignant bone tumor in the epi-meta-diaphyseal of left ulnar bone extended into proximal humeroulnar and radioulnar joints without neurovascular involvement (Fig. 2). Core biopsy showed a Giant Cell containing lesion.

**Fig. 3:**
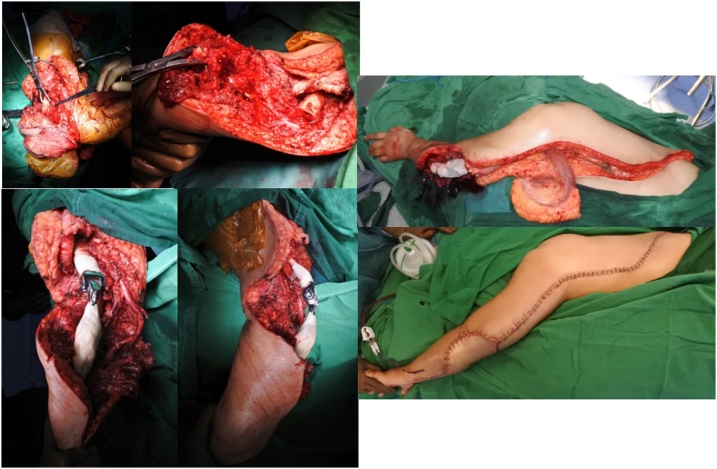
Intraoperative procedure. (A) Exposing the tumor followed by wide excision of the proximal ulnar bone. (B) Elbow arthroplasty reconstruction. (C) Defect closure with latissimus dorsi flap.

**Fig. 4:**
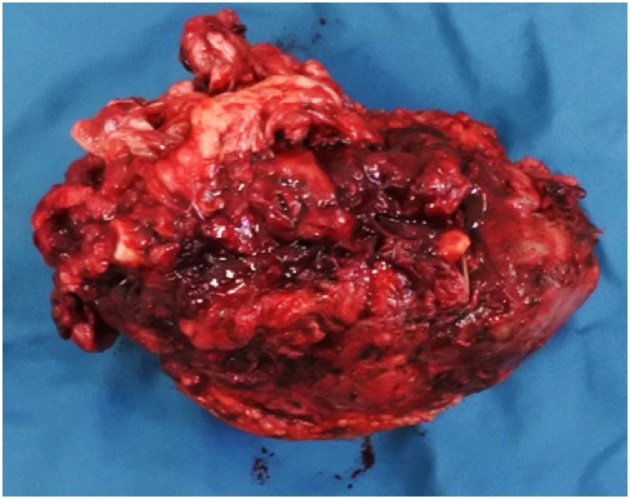
Macroscopic of tumor. Tumor size 10.3 × 6.8 × 5.2 cm, red- white colours, with solid palpable, and necrotic in the borders area.

We brought this case to a Clinico Pathological Conference (CPC) to confirm the diagnosis and decide further management. Our discussion resulted in Giant Cell Tumor as the diagnosis and wide excision and elbow arthroplasty as the treatment. We performed a latissimus dorsi flap to enclose the relatively wide excised area (Fig. 3). The procedure was done without any intraoperative complication. Resected tumor (Fig. 4) was sent for histology confirmation.

**Fig. 5:**
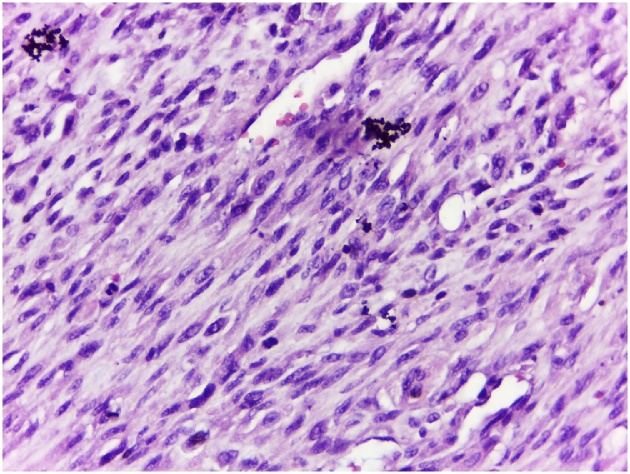
Histology of resected tumor. Osteoclastic giant cells surrounded by oval or spindle mononuclear cells in a fibrovascular stroma and lack of osteoid matrix.

The histology of resected tumor was evenly distributed osteoclastic giant cells surrounded by oval or spindle mononuclear cells in a fibrovascular stroma (Fig. 5). Most stromal cells showed mild nuclear pleomorphism and infrequent mitosis but, focally, they possessed moderately to markedly pleomorphic nuclei; some showed prominent nucleoli. There were focal irregular or lacelike osteoid deposits around stromal cells. It made us consider a malignant probability and planned to start neoadjuvant chemotherapy in the second CPC, one month after the first conference. Ki67 staining was also done which confirm the diagnosis of giant cell-rich osteosarcoma (GCRO) later in the third CPC.

## 3 Discussion

Giant cell rich osteosarcoma (GCRO) is an uncommon variant which constitutes about 3% of osteosarcoma. It is characterized by an abundance of osteoclastic giant cells and lack of tumor osteoid. This leads to its confusion with a giant cell tumor (GCT), especially when it occurs in an epiphyseal location. [12] GCRO mimics GCT radiological features and makes it hard to differentiate them [12].

Involvement of forearm in conventional osteosarcoma is extremely rare. Primary tumor of the ulna accounted for less than 1% of all lesions and less than 2% of all bone lesions. Exner et al. reported that the ulna was the primary site for malignant bone tumors in three cases out of more than 2000 neoplasms and tumor-like bone lesions in the Balgrist tumor registry.^1^ This was the epidemiologic reason why the osteosarcoma was not the first diagnosis of our case.

Our case radiographic appearances showed expansile osteolytic lesions accompanied by cortical erosion, periosteal reaction, and absence of osteoblastic areas while the MRI examination revealed internal septa with multiloculations and multiple fluid levels. Such features are similar in both GCRO and GCT with regard to gross and histomorphology. While the stromal and giant cell, fibrohistiocytic and aneurysmal-cystic patterns may be seen in GCRO and GCT, the osteoblastoma-like and parosteal and fibrous dysplasia-like patterns are not found in the GCT. The most important is lace-like osteoid deposition and permeative infiltration that distinguish GCRO from GCT. Finally, the stromal and giant cell pattern exceeding 50% establishes the diagnosis of GCRO. Ki67 proliferative index is high in all GCRO, over 30% in the stromal and giant cell pattern which also found in our case. Some author suspecting this high proliferative index as an aggressive potential of tumor [12].

**Fig. 6:**
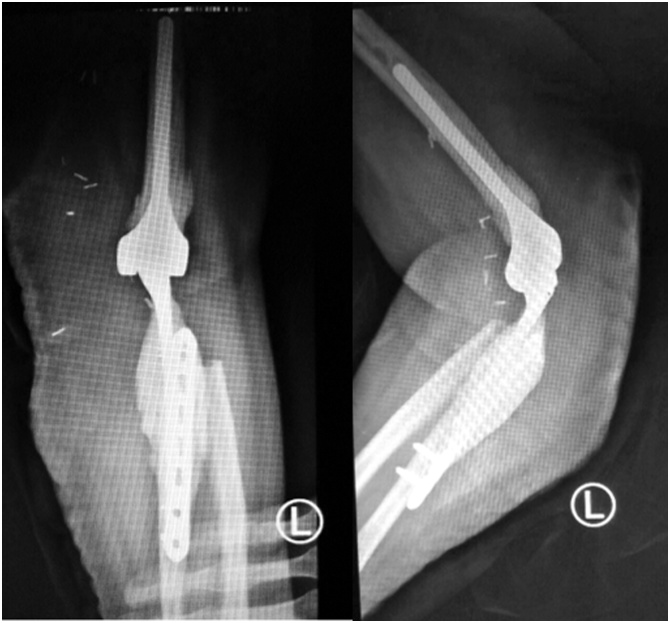
Post Operative Xray. Showing elbow arthroplasty, ulnar bone was reconstructed using a 3.5 locking plate.

Being a very rare location of the osteosarcoma, the management poses a special challenge. They are the surgical technique, which is not well described, and the implant that need to be custom made. The definitive treatment should be radical en bloc resection including surrounding intact tissue, to guarantee that no recurrence will occur and avoid the risk of massive bleeding. After wide excision, a large defect was a challenge in restoring the biomechanics of the adjacent joints. Elbow arthroplasty have shown to provide better long-term outcomes and are commonly used to get better motion. Because of a large defect in the bone and soft tissue, we used a locking plate for connecting the ulnar bone (Fig. 6) and latissimus dorsi flap for the soft tissue closure [4].

## 4 Conclusion

Giant cell rich osteosarcoma is difficult to diagnose because its histology and radiologic feature mimic a benign giant cell tumor. Our case was a rare type of osteosarcoma occurs in a rarely found proximal of ulnar bone. Thus, make this case is very challenging in both diagnosis and the treatment. We performed an elbow arthroplasty followed by latissimus dorsi flap without neither intraoperative nor postoperative complication. It is required to observe the patient for a longer period to detect a recurrence and metastasis.

## Conflicts of interest

The authors have no ethical conflicts to disclose.

## Sources of funding

There is no sources of funding sponsor in this manuscript.

## Ethical approval

Approval has been given by the ethics committee of the Faculty of Medicine, Universitas Indonesia. The reference number is 073/UN2.F1/ETIK/2019.

## Consent

Written informed consent was obtained from the patient for publication of this case report and accompanying images. A copy of the written consent is available for review by the Editor-in-Chief of this journal on request.

## Author contribution

1 Sigit Daru Cahayadi, MD. Contributed as making the case report, funding, and final approval of manuscript.
2 Ajiantoro, MD. Contributed as making case report, collecting the data, and writing manuscript.

Bram Swandika, MD. Contributed as making case report, and writing manuscript.

## Registration of research studies

N/A.

## Guarantor

Sigit Daru Cahayadi, MD.

## Provenance and peer review

Not commissioned externally peer reviewed.
